# Drug-induced liver injury due to varenicline: a case report

**DOI:** 10.1186/1471-230X-12-65

**Published:** 2012-06-08

**Authors:** David Sprague, Kiran Bambha

**Affiliations:** 1 Department of Internal Medicine, University of Colorado Denver, Aurora, CO, USA; 2 Division of Gastroenterology and Hepatology, University of Colorado Denver, Aurora, CO, USA

**Keywords:** Drug induced liver injury, Varenicline, Idiosyncratic

## Abstract

**Background:**

Liver injury due to prescription and nonprescription medications is an expanding public health concern in the United States, with drug-induced liver injury (DILI) being the single most common reason for regulatory actions instituted by the Food and Drug Administration against certain medications and supplements.

**Case presentation:**

A 69-year-old Latino man was referred to Hepatology Clinic for urgent evaluation of new onset jaundice, nausea and fatigue associated with a >40-fold increase in his transaminase levels and elevated INR and alkaline phosphatase. The patient had received a new prescription for varenicline to aid with smoking cessation approximately 3 weeks prior to his evaluation in Hepatology Clinic. Within 5 days of starting the varenicline, the patient developed new onset of nausea, vomiting, malaise and deep jaundice. The varenicline was discontinued on day 5 by the patient. Hepatologic evaluation revealed no evidence of acute viral hepatitis, autoimmune, metabolic or alcohol-related liver disorders. The patient’s past medical history was notable, however, for chronic hepatitis C. His liver enzymes and synthetic function completely normalized 9 weeks after discontinuation of the varenicline.

**Conclusion:**

This report represents the second documented cases of drug-induced liver injury related to varenicline therapy, highlighting the need for clinician awareness regarding potential hepatotoxicity of varenicline, particularly among patients with pre-existing liver disease.

## Background

Drug-induced liver injury (DILI) is an adverse drug reaction that results in acute liver injury ranging in severity from mild, asymptomatic elevations in liver biochemistries to acute liver failure culminating in death or liver transplantation. Liver injury due to prescription and nonprescription medications is an expanding public health concern in the United States, with DILI being the single most common reason for regulatory actions instituted by the Food and Drug Administration against certain medications and supplements [1]. The incidence of DILI has been challenging to quantify due to it being a relatively uncommon clinical entity, and also due to under-recognition of the disorder; however, the worldwide annual incidence rate of DILI is estimated to be approximately 13.9-24.0 cases per 100,000 inhabitants, with DILI accounting for 3-9 % of all adverse drug reactions reported to health authorities [2-4]. DILI, whether acetaminophen-induced or idiosyncratic, has been implicated in up to 50 % of acute liver failure cases in the United States. Depending on the inciting pharmacologic agent, DILI has been associated with a case-fatality rate of 10-50 %. Thus, given the continually expanding market of prescription and nonprescription medications and supplements, DILI will remain a clinically important etiology of acute liver injury, making timely and accurate reporting of DILI important for the early detection and awareness of drug-induced hepatotoxicity.

We present a case of hepatotoxicity associated with varenicline use. Varenicline is a partial agonist at the α4β2 nicotinic acetylcholine receptors and is supplied as an orally administered tablet. It may be used alone or in combination with other smoking cessation agents as a first-line therapy for smoking cessation. Commonly reported side effects to varenicline include nausea, vomiting, neuropsychiatric and sleep disturbances. To date, there has been only one published case report of varenicline-associated hepatotoxicity [5].

## Case presentation

A 69-year-old Latino man was seen for urgent consultation in the outpatient Hepatology Clinic for evaluation of new onset jaundice and substantially elevated liver enzymes associated with nausea and extreme malaise that developed three weeks prior to presentation. The patient’s past medical history was notable for type 2 diabetes mellitus, hypertension and tobacco abuse. He had no known diagnosis of chronic liver disease prior to the onset this illness. The patient was a widower and retired foreman of a starch plant who endorsed a greater than 25 pack-year smoking history and an alcohol history of 1–2 cans of beer per week, on average. His last drink was one month prior to the onset of the current illness. He had a history of tattooing as a teenager, but denied history of recreational drug use and denied any non-prescription or herbal supplement use. His family history was significant for both diabetes and coronary artery disease; there was no known family history of liver disease.

The patient was in his usual state of health and feeling quite well when he presented to his primary care provider for a routine health maintenance check-up approximately 3 weeks prior to presentation in Hepatology Clinic. At that time, the patient was counseled regarding the health benefits of smoking cessation, particularly given his known history of diabetes and hypertension. The patient was receptive to smoking cessation intervention and was prescribed varenicline 0.5 mg orally once daily, which he began taking four weeks prior to his presentation in Hepatology Clinic. The patient also continued on his long-term chronic medications that included: metformin 850 mg orally twice daily, aspirin 81 mg orally daily, valsartan 80 mg orally daily, and sildenafil 50 mg orally daily as needed. Five days after starting the varenicline, the patient developed new onset nausea, vomiting, decreased appetite, weight loss and extreme malaise. The patient’s son noticed yellowing of his father’s eyes and prompted him to seek immediate medical attention. Given these symptoms, the patient discontinued the varenicline on his own at day 5. The patient was seen by his primary care provider approximately two-and-a-half weeks after starting the varenicline. Liver chemistries performed at that time revealed the following: aspartate aminotransferase (AST) = 1191 U/L, alanine aminotransferase (ALT) = 1592 U/L, alkaline phosphatase (Alk Phos) = 254 U/L, total bilirubin (TBili) = 12.0 mg/dL, INR = 1.3, platelets = 289 x 10^6^ cells/mL and serum creatinine = 1.0 mg/dL. His INR was mildly elevated at 1.3 and serum albumin was normal at 4.0 g/dL. Hemoglobin and white blood cell counts were within normal limits. No peripheral eosinophilia was noted. Acetaminophen and alcohol levels were undetectable. Viral hepatitis serologies were negative for hepatitis A (HAV) IgM, hepatitis B (HBV) sAg, and HBc IgM. Hepatitis C (HCV) Ab was noted to be positive. As the patient had no known antecedent history of abnormal liver enzymes, he had never previously been tested for HCV. A CT scan of the abdomen revealed a normal appearing liver without any evidence for cirrhosis or biliary ductal dilation or hepatic masses. Hepatic artery, hepatic veins and portal veins were all patent. Magnetic resonance cholangiopancreatography (MRCP) was also performed and was negative.

 Due to the significant hepatitis and hyperbilirubinemia, the patient was seen urgently in our Outpatient Hepatology Clinic, marking approximately three weeks after the onset of symptoms. At the time of the visit, the patient continued to complain of significant nausea, vomiting, poor appetite, 10-pound weight loss, dark urine and persistent jaundice. He continued to abstain from the use of varenicline. On physical examination, the patient was 5’6” tall and weighed 125 pounds. He had prominent bilateral scleral icterus and jaundiced skin. No skin rashes or lymphadenopathy were noted. Abdominal exam was unremarkable, without distention, tenderness or hepatosplenomegaly. There were no classical stigmata of chronic liver disease. Repeat laboratory studies were performed at the time of the Hepatology consultation and revealed the following: AST = 334 U/L, ALT = 515 U/L, T Bili = 18.1 mg/dL, INR = 1.2, Albumin = 3.3 g/dL and Alk Phos = 137 U/L. HCV genotype was 1a with HCV RNA level of 55,100 IU/mL. HIV testing was negative. Further laboratory testing demonstrated that the patient was negative for HBV DNA, anti-nuclear antibody, anti-smooth muscle antibody and anti-mitochrondrial antibody. Immunoglobulin and thyroid stimulating hormone levels were normal and alpha-1-antitrypsin phenotype was MM. Testing for hemochromatosis and celiac sprue were negative. Evaluation for other acute viral infections, including herpes simplex (HSV)-1 and −2, Epstein Barr virus (EBV) and Cytomegalovirus (CMV) were negative.

**Figure 1:**
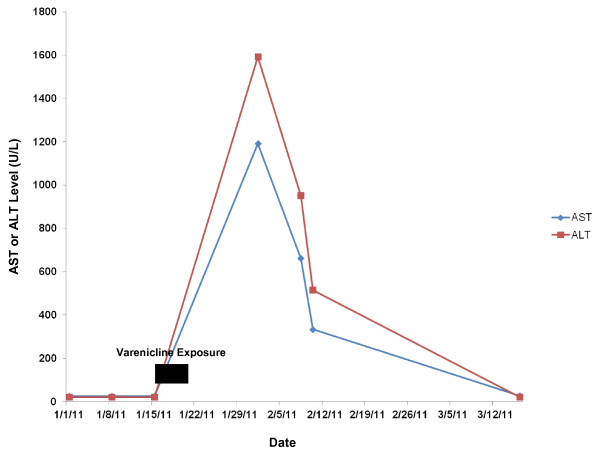
Evolution of aspartate (AST) and alanine (ALT) aminotransferase levels with varenicline exposure and discontinuation.

**Figure 2:**
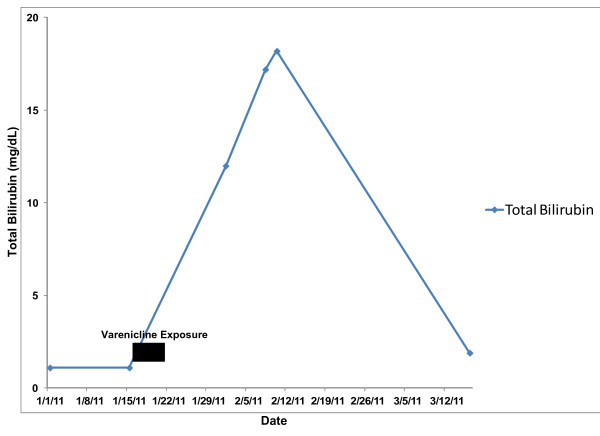
Evolution of total bilirubin (T Bili) levels with varenicline exposure and discontinuation.

Approximately two months after stopping the varenicline, the patient’s AST, ALT and Alk Phos had normalized with near-normalization of the T Bili. (Figures 1 and 2) He reported a substantial improvement in his health status and normalization of his activity levels with resolution of the nausea, vomiting, fatigue and jaundice, and a return of his appetite. A percutaneous liver biopsy was performed at this point with the goal of grading and staging the patient’s HCV and demonstrated changes consistent with chronic HCV, grade 2, stage 2 disease. Also noted, however, were focal lobular aggregates of pigmented Kupffer cells suggestive of more recent and significant hepatocyte injury, correlating well with the patient’s clinical history. The patient was instructed never to rechallenge himself with varenicline and to report it as a medication adverse reaction in the future.

## Conclusion

Acute hepatic injury caused by drugs, both prescription and non-prescription, is termed drug-induced liver injury (DILI). DILI may range in severity from mild, asymptomatic, elevations in liver enzymes to severe hepatic injury culminating in acute liver failure resulting in either death or liver transplantation. There are currently just under a thousand drugs that have been suspected of causing DILI. DILI may be predictable and dose-dependent, as classically exemplified by acetaminophen toxicity, or may be idiosyncratic [6-8]. Idiosyncratic drug-induced liver injury, as exemplified in the current case report of varenicline, represents a complex interaction between an individual’s unique metabolic and genetic characteristics and the drug [9,10]. Idiosyncratic drug-induced liver injury is unpredictable and not directly related to the dose, duration or route of administration of the drug. The majority of suspect drugs involved in cases of idiosyncratic DILI were not associated with hepatotoxicity in the preclinical or early clinical testing of the drug. This is likely the result of several factors including: the relative rarity of severe idiosyncratic DILI, which has a reported incidence between 1 in 10,000 and 1 in 100,000 patients exposed; and the relatively small size of most clinical drug trials with respect to detecting such an uncommon event as severe DILI. This scenario is best exemplified by troglitazone (Rezulin®), which was ultimately withdrawn from the market in 2000 due to multiple reported cases of acute liver failure [11]. Therefore, it may not be until a drug comes to market and thousands of patients are exposed to the drug that cases of DILI begin to be recognized. This phenomenon also underscores the importance of clinical providers reporting cases of suspected DILI to the Food and Drug Administration.

The diagnosis of DILI can be challenging as there are no specific diagnostic tests and DILI is essentially a diagnosis of exclusion of other etiologies of acute liver injury. The diagnosis may be even more challenging in the setting of known chronic liver disease and in patients taking multiple potentially hepatotoxic drugs. The evaluation of the patient with suspected DILI should include laboratory testing and clinical assessment for an array of etiologies of abnormal liver function, including: viral hepatitis, excessive alcohol use, autoimmune liver disease, vascular disorders of the liver and metabolic liver diseases. Evidence for hepatic ischemia and biliary disease should also be pursued.

In the case report presented here, DILI due to varenicline was the most likely etiology of our patient’s acute liver injury. Extensive testing for other causes of acute liver injury was negative and the patient did not endorse signs, symptoms or imaging findings suggestive of outflow obstruction, hepatic ischemia or biliary obstruction. Although the patient was discovered to be HCV Ab positive with detectable HCV RNA, this was very unlikely to be an acute infection given his lack of recent risk factors for HCV acquisition and his liver biopsy demonstrating stage 2 fibrosis.

DILI may be classified into three categories based upon the pattern of liver injury observed: (1) acute hepatocellular injury; (2) cholestatic injury; and (3) mixed liver injury [12]. Hepatocellular injury is due to substantial damage to the hepatocytes and is characterized by elevations in the serum AST and ALT levels, with lesser elevations in the Alk Phos level. Cholestatic injury results from either direct injury to the biliary epithelium or injury to the molecular processes involved in bilirubin metabolism and secretion and is characterized by disproportionately elevated Alk Phos and T Bili levels. Mixed liver injury has both hepatocellular and cholestatic features. Notably, T Bili may be substantially elevated in any of these three categories of liver injury and generally signifies more severe liver injury. In the case of our patient, he demonstrated a predominantly hepatocellular injury that occurred with a substantially elevated T Bili and a mild elevation in his INR, consistent with a moderately severe liver injury.

Assessing causality in suspected cases of DILI remains a challenge. Two algorithms specifically designed for DILI causality assessment, the Roussel Uclaf Causality Assessment Model [RUCAM] and the Maria & Victorino [M&V] Scale, have been published and, although they are not widely used in the clinical setting, they do offer useful frameworks for thinking about the patient with suspected DILI [13,14]. The RUCAM assesses several different domains, including: chronological relationship between the drug exposure and hepatic injury, risk factors, concomitant drug use, evaluation for other etiologies of liver injury, pre-existing data regarding the hepatotoxic potential of the suspected drug, and response of the patient to clinical rechallenge with the suspected drug. Using the RUCAM, a score ranging from −9 to 14 is generated and patients are grouped into categories of: DILI excluded; DILI unlikely; DILI possible; DILI probable; and DILI highly probable. Applying the RUCAM causality assessment tool to our case results in varenicline being rated as the “probable” cause of DILI. The M&V scale was developed in an attempt to simplify the scoring system for DILI. The M&V scale focuses on: the time course of the drug exposure and its relationship to the liver injury, exclusion of completing causes of liver injury, pre-existing data implicating the suspected drug in causing liver injury, and response of the patient to rechallenge with the suspected drug. Using the M&V scoring system, a score ranging from −8 to 20 is generated and patients are grouped into five categories rating the likelihood of DILI: DILI excluded; DILI unlikely; DILI possible; DILI probable; DILI definite. In applying the M&V scale to our case, varenicline is categorized as a “possible” cause of DILI. It is notable that both the RUCAM and the M&V scales are heavily weighted based upon whether rechallenge with an offending medication results in repeat DILI. Rechallenge with varenicline was not attempted in our patient due to concerns for his safety.

To date, there has been one previously published case of DILI associated with varenicline. In 2009, Franck and Sliter described the case of a 53 year old man with HCV and alcohol-induced cirrhosis who developed a predominant hepatitis after being initiated on varenicline for smoking cessation while awaiting liver transplantation. The patient developed a significant hepatitis within 4 weeks of beginning varenicline therapy that resolved after discontinuing the medication. In contrast to our patient, the previously published case involved a patient with cirrhosis and did not demonstrate a substantial rise in the bilirubin level. Data on the INR were not reported.

Pharmacoepidemiological studies have suggested that certain pre-existing disease states and certain physiochemical properties of certain classes of drugs might increase the risk of idiosyncratic DILI, although the data regarding this issue are equivocal [15]. Interestingly, the published pharmacokinetics of varenicline demonstrate that it is not substantially metabolized by the liver and is eliminated via glomerular filtration. Therefore, there are currently no recommended dose adjustments or precautions for varenicline in patients with liver disease.

While the presence of underlying liver disease and HCV in the present case and that reported previously by Franck and Sliter may be coincidental, it is also of potential clinical relevance. Both chronic liver disease and smoking are prevalent in the U.S. adult population. Currently, 19.6 % of the U.S. adult population smokes [16]. And among individuals with HCV this rate is even higher, estimated at 58 % [17]. As Franck and Sliter note in their case report, it is commonly recommended by liver transplant programs that patients being considered for liver transplantation be encouraged to abstain from smoking. Because sustained-release bupropion is recommended for use with extreme caution in cirrhotic patients, varenicline represents the only other non-nicotine replacement pharmacologic intervention for smoking cessation in this population. Therefore patients may be at risk for DILI if an under-explored association between hepatocellular dysfunction and varenicline in the setting of HCV, or any chronic liver disease, exists. The case presented here has been reported to MedWatch. Future cases of suspected varenicline-induced hepatotoxicity should be reported and efforts to delineate the mechanism of injury undertaken.

## Consent

Written informed consent was obtained from the patient for publication of this case report. A copy of the written consent is available for review by the Editor-in-Chief of this journal.

## Abbreviations

DILI, Drug Induced Liver Injury; AST, Aspartate Aminotransferase; ALT, Alanine Aminotransferase; Alk Phos, Alkaline Phosphatase; T Bili, Total Bilirubin; HAV, Hepatitis A Virus; HBV, Hepatitis B Virus; HBc, Hepatitis B core; HCV, Hepatitis C Virus; HSV, Herpes Simplex Virus; EBV, Epstein Barr Virus; CMV, Cytomegalovirus; RUCAM, Roussel Uclaf Causality Assessment Model; M&V Scale, Maria & Victorino Scale.

## Competing interests

The authors declare that they have no competing interests.

## Authors’ contributions

DS collected the references and wrote the manuscript. KB initiated the paper, helped to incorporate the clinical data into the manuscript and mentored the writing and completed the revisions of the manuscript. All authors report no conflicts of interest and approved the final manuscript.

## Pre-publication history

The pre-publication history for this paper can be accessed here:

http://www.biomedcentral.com/1471-230X/12/65/prepub
